# Large-scale fabrication of nanopatterned sapphire substrates by annealing of patterned Al thin films by soft UV-nanoimprint lithography

**DOI:** 10.1186/1556-276X-8-472

**Published:** 2013-11-11

**Authors:** Lin Cui, Jie-Cai Han, Gui-Gen Wang, Hua-Yu Zhang, Rui Sun, Ling-Hua Li

**Affiliations:** 1 Shenzhen Graduate School, Harbin Institute of Technology, Shenzhen 518055, People's Republic of China; 2 Center for Composite Materials, Harbin Institute of Technology, Harbin 150080, People's Republic of China

**Keywords:** Al, Annealing, Patterned sapphire substrates, UV-nanoimprint lithography, Reactive ion etching

## Abstract

Large-scale nanopatterned sapphire substrates were fabricated by annealing of patterned Al thin films. Patterned Al thin films were obtained by soft UV-nanoimprint lithography and reactive ion etching. The soft mold with 550-nm-wide lines separated by 250-nm space was composed of the toluene-diluted polydimethylsiloxane (PDMS) layer supported by the soft PDMS. Patterned Al thin films were subsequently subjected to dual-stage annealing due to the melting temperature of Al thin films (660°C). The first comprised a low-temperature oxidation anneal at 450°C for 24 h. This was followed by a high-temperature annealing in the range of 1,000°C and 1,200°C for 1 h to induce growth of the underlying sapphire single crystal to consume the oxide layer. The SEM results indicate that the patterns were retained on sapphire substrates after high-temperature annealing at less than 1,200°C. Finally, large-scale nanopatterned sapphire substrates were successfully fabricated by annealing of patterned Al thin films for 24 h at 450°C and 1 h at 1,000°C by soft UV-nanoimprint lithography.

## Background

High output power GaN-based light-emitting diodes (LEDs) attract much attention because of their various applications in traffic signals, full-color displays, backlight in liquid crystal displays, solid-state lighting, and so forth [1]. At present, because of the difficulty of obtaining high-quality and reasonable-cost GaN substrates, sapphire is most commonly used as the substrate for LEDs due to its high-temperature stability and physical robustness. However, owing to the large lattice mismatch and thermal expansion between the epitaxial GaN film and the underneath sapphire substrate, high threading dislocation densities with the order of 10^9^ to 10^10^ cm^−2^ and deterioration of the electrical and optical properties, therefore, lead to poorer internal quantum efficiency (*η*_int_) and reliability [2,3]. On the other hand, the refractive index of nitride films (*n* = 2.5) is higher than that of sapphire substrates (*n* = 1.78) and air (*n* = 1). The critical angle of the escape cone is about 23°, which indicates that only about 4 % of the generated light in the active layer can be extracted from the surface and mostly absorbed by the electrode at each reflection and gradually disappears due to total internal reflection, and is then converted to heat [4].

Many different growth approaches have been proposed to improve the performances of epitaxial GaN films; the epitaxial lateral overgrowth (ELOG) technique is known to significantly reduce threading dislocations effectively [5,6]. However, this approach is a time-consuming process and often requires a two-step growth procedure and introduces uninterrupted dopants or contaminations. Recently, it has been reported that one can not only reduce the threading dislocation density in GaN films but also enhance the light extraction efficiency by using a patterned sapphire substrate (PSS) [7,8]. However, the dimension of PSS with grooves or other patterns is usually in micron-scale range. Theoretical and experimental studies indicate that a further reduction in defect density is possible if the dimension of the lateral overgrowth patterns is extended to nanoscale range [9-11].

Many articles reported that sapphire substrates are nanopatterned by dry etching and wet etching. It is known that sapphire is chemically inert and highly resistive to acids at room temperature. Thus, it is extremely difficult to etch sapphire substrates using a chemical solution at room temperature. Compared with wet etching, dry etching can provide us an anisotropic profile and a reasonably fast etching rate [12], but dry-etched substrates will be inevitably damaged, and the device performance is compromised [13]. To resolve the problem in dry and wet etching processes, Cui et al. [14] have reported the effect of exposure parameters and annealing on the structure and morphological properties of nanopatterned sapphire substrates prepared by solid-state reaction and e-beam lithography. However, e-beam lithography is not a cost-effective solution due to expensive equipment and low efficiency for the fabrication of large-area patterns. UV-nanoimprint lithography (UV-NIL) has been gaining attention in the semiconductor industry as one of the candidates for the next-generation manufacturing technology of low cost, wide distribution, and high patterning resolution [15,16]. Moreover, UV-NIL using soft polydimethylsiloxane (PDMS) mold has advantages over conventional methods for patterning of imprinted area, surface roughness, and curvature of substrate [17]. Therefore, in this study, large-scale nanopatterned sapphire substrates (NPSS) were fabricated by dual-stage annealing of patterned Al thin films prepared by soft UV-NIL and reactive ion etching (RIE).

## Methods

**Figure 1:**
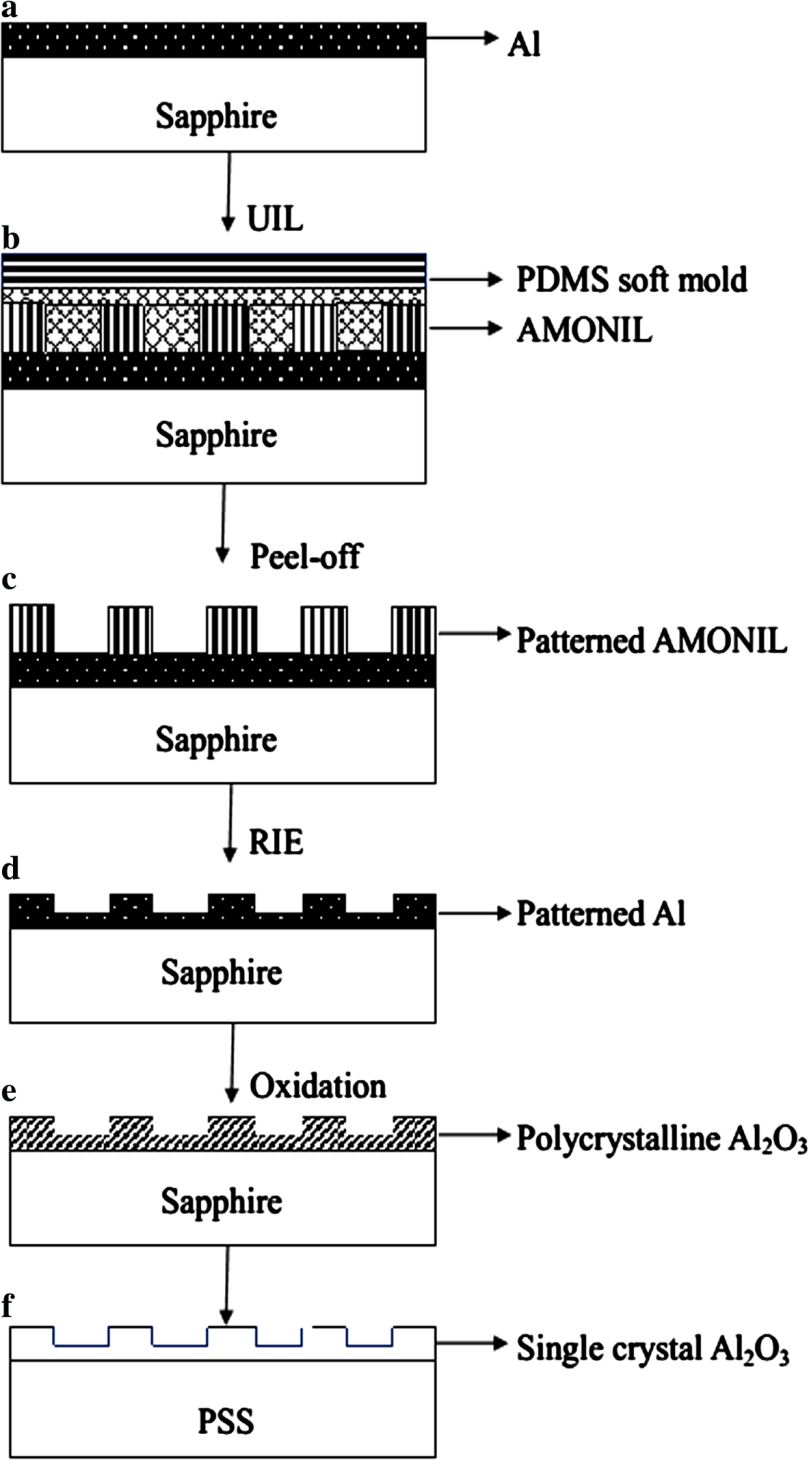
Schematic diagram showing processing steps in the generation of large-scale NPSS.

The process of large-scale NPSS consisted of the following steps (Figure 1): (a) 150-nm Al thin films were deposited on sapphire (0001) substrates, (b) UV-NIL resist, (c) peeled off PDMS soft mold, (d) patterned Al thin films were obtained with the RIE process, (e) oxide-patterned Al thin films, and (f) grain growth of patterned polycrystalline alumina thin films.

High-purity Al thin films were deposited on sapphire (0001) substrates by direct current (DC) sputtering in a JGP-450a magnetron sputtering system. Prior to deposition, the sapphire substrates were ultrasonically cleaned with acetone for 10 min and alcohol for another 10 min, rinsed with deionized water, and then dried withN_2_. A 99.999 % pure Al target of 2-in. diameter was used, and the plasma of Ar (99.999 %) was used for sputtering. The distance between the target and substrate was 70 mm. The base pressure was less than 8 × 10^−5^ Pa. Deposition was carried out at a working pressure of 0.2 Pa after presputtering with Ar for 10 min. When the chamber pressure was stabilized, the DC generator was set to 60 W. The deposition rate utilized was 18 nm/min.

**Figure 2:**
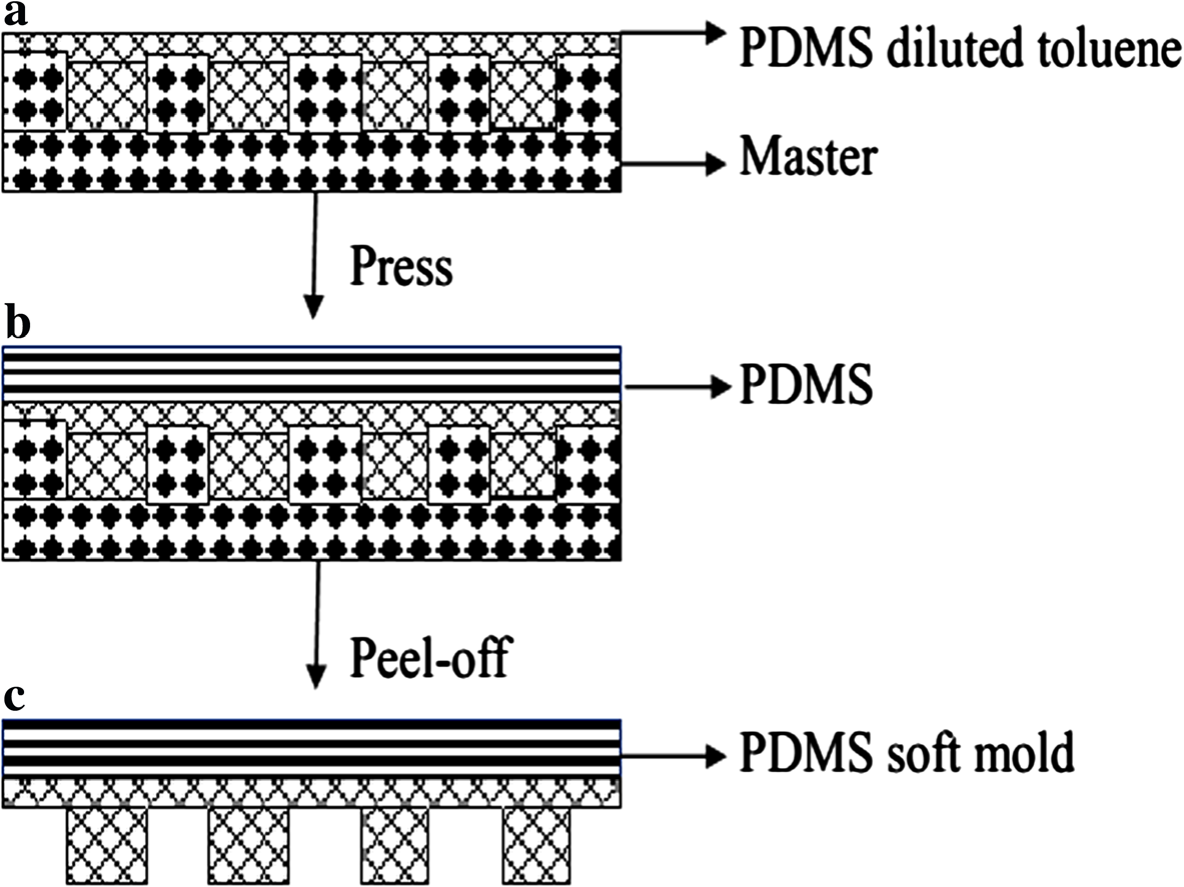
Schematic illustration of soft PDMS mold based on quartz master mold.

The 2-in. quartz master mold with 250-nm-wide and 150-nm-long lines separated by 550-nm space was fabricated by laser interference lithography and RIE. Prior to replication of soft PDMS mold, the quartz master self-assembled an anti-adhesive monolayer (1*H*,1*H*,2*H*,2*H*-perfluorodecyltrichloro-silane (FDTS)) by vapor phase deposition to yield a low surface free energy, which is required to detach easily the quartz master and soft PDMS. Figure 2 shows the schematic illustration of the soft PDMS mold based on the quartz master mold. In this paper, we designed a scheme of replication based on the quartz master mold: PDMS was diluted with toluene (60 wt.%) to decrease the viscosity, since the modification of the PDMS ensures high fidelity of pattern features by UV-NIL [18]. The degassed modified PDMS was spin-coated at 3,000 rpm for 30 s on the quartz master mold. After degassing, the quartz master mold with a uniform layer was cured at 120°C for 15 min. Then the degassed PDMS prepolymer (Sylgard 184, Dow Corning, Midland, MI, USA) and its curing agent (1:10 weight) were carefully poured onto the surface, followed by curing at 100°C for 30 min. Afterwards, the 2-in. soft mold, the modified PDMS supported by thick, flexible PDMS layer, was peeled off from the quartz master mold.

After the deposition of Al thin films, the 220-nm-thick UV-curable resin AMONIL-MMS4 (AMO GmbH, Aachen, Germany) was spin-coated at a speed of 3,000 rpm for 30 s onto 150-nm-thick Al thin films. At 100°C, the AMONIL-MMS4 was prebaked on a hot plate. The UV-NIL was performed on an EVG620 (EVG Group, Schärding, Austria). The nanoimprint pressure is 3 × 10^4^ Pa, and the hold time of UV exposure is 90 s. The residual polymer layer was then removed by RIE (CRIE-100, AST, Hsinchu County, Taiwan). The O_2_ gas flow rate, working pressure, radio-frequency (RF) power, DC bias voltage, and etch time were maintained at 200 sccm, 13 Pa, 50 W, −200 V, and 120 s, respectively. The patterns were subsequently transferred into Al thin films by RIE. The BCl_3_ and Cl_2_ gas flow rates, working pressure, RF power, DC bias voltage, and etch time were maintained at 100 and 25 sccm, 1 Pa, 600 W, −200 V, and 90 s, respectively.

The nanopatterned Al thin films were subsequently subjected to dual-stage annealing. Our experimental results reveal that the hillock formation on Al thin films was minimized with an oxidation anneal at 450°C [14]. Therefore, the first comprised an oxidation anneal, where the annealing temperature was 450°C for 24 h. The temperature ramp rate was 10°C/min. This was followed by a high-temperature annealing in the range of 1,000°C to 1,200°C for 1 h. The temperature ramp rate was 10°C/min up to 800°C and then 5°C/min thereafter. All annealing treatments were carried out in air in a box furnace with the substrates contained in a high-purity alumina crucible. In this study, the surface morphology was examined using an atomic force microscope (AFM; Veeco DID3100, Plainview, NY, USA) and scanning electron microscope (SEM; Hitachi S-4700, Tokyo, Japan).

## Results and discussion

**Figure 3:**
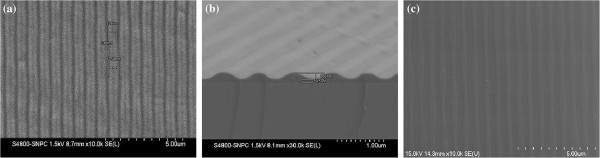
**SEM images of the morphology of PDMS soft mold molding.** From the quartz master (a), patterned AMONIL-MMS4 (b), and patterned Al thin films obtained by the UV-NIL and RIE (c).

Top-view SEM micrograph of soft mold (PDMS diluted with toluene) molding from the quartz master is shown in Figure 3a. As shown in Figure 3a, the patterned PDMS with 550-nm-wide lines separated by 250-nm space were obtained on the surface. The result of the UV curing imprinted pattern used by the replicated soft PDMS mold on the quartz master is shown in Figure 3b. It is easily seen that the patterned AMONIL-MMS4 with 250-nm-wide and 120-nm-long lines separated by 550-nm space was obtained on the Al thin film surface, which is coincident with that of the quartz master. The residual polymer layer with 60-nm thickness was removed by RIE. The patterns were subsequently transferred into Al thin films by RIE. Top-view SEM micrograph of patterned Al thin films obtained by the UV-NIL and RIE is shown in Figure 3c. As shown in Figure 3c, the patterned Al thin films with 250-nm-wide lines separated by 550-nm space were obtained on the sapphire surface, which is coincident with that of the quartz master.

**Figure 4:**
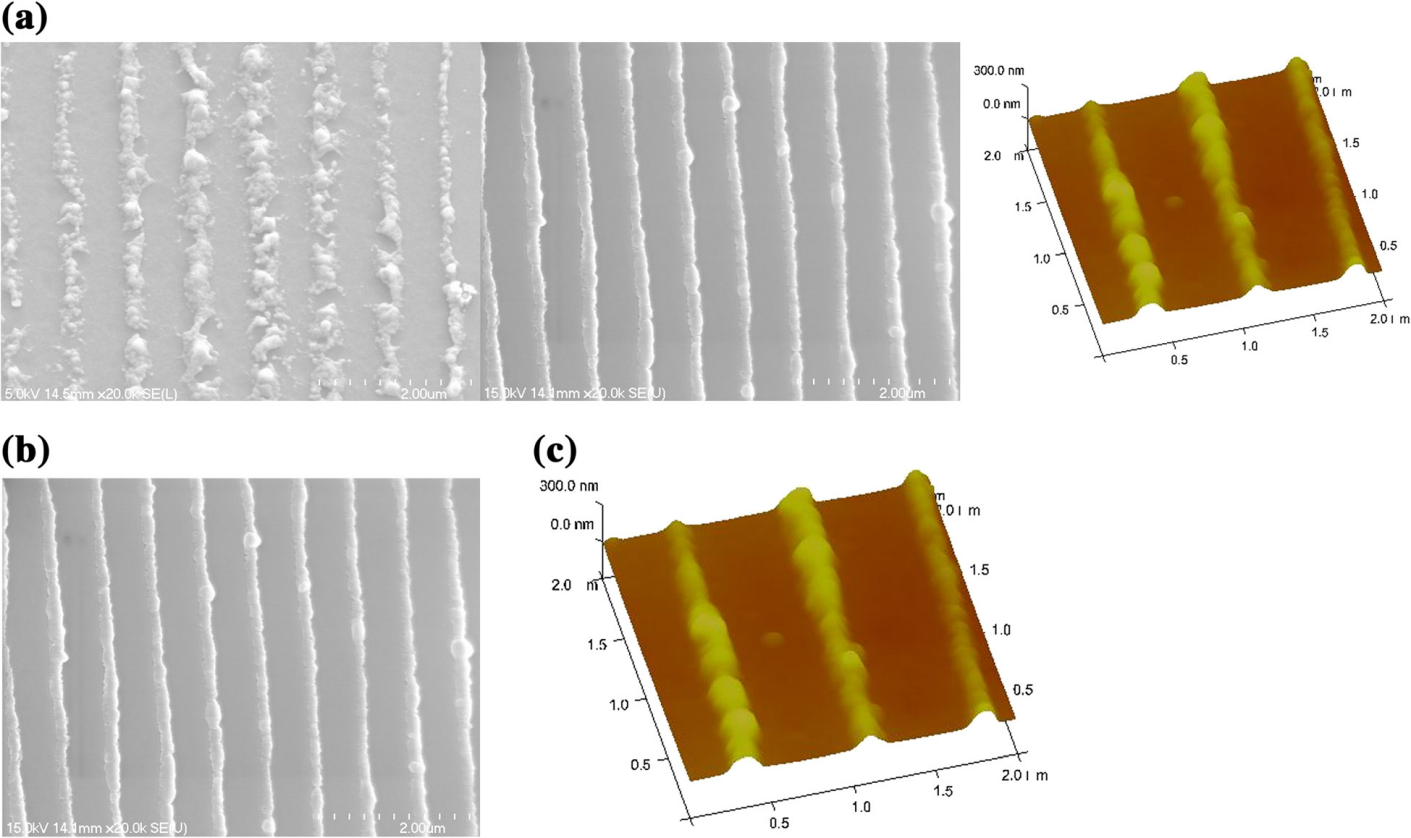
**SEM and AFM images of Al patterns after annealing.** SEM images of the morphology of the Al patterns on sapphire substrates after annealing for 24 h at 450 °C and 1 h at 1,200°C **(a)** and 1,000°C **(b)**. AFM image of Al patterns after dual-stage annealing for 24 h at 450°C and 1 h at 1,000°C **(c)**.

Dramatic changes in the pattern morphology were observed following high-temperature annealing applied to induce grain growth of the sapphire. Figure 4a shows a SEM image of the morphology of the patterned surface after annealing for 24 h at 450°C and 1 h at 1,200°C. For nanopatterned Al thin films that subsequently experienced an annealing temperature of 1,200°C, it was found that smoothing and coalescence of the line features had occurred to such an extent that the patterning was no longer discernible. The phenomenon of surface diffusion-driven smoothing of surface features is well established in the literature [19-22] and occurs due to surface energy considerations [23,24]. The kinetics of the smoothing of the line patterns can be used to derive information on the diffusion mechanism. Therefore, for the successful fabrication of NPSS, the relative kinetics of smoothing versus grain growth of the underlying sapphire is critical. Fortunately, for high-temperature annealing at 1,000°C and 1,100°C, the patterns were retained on sapphire substrates. Figure 4b shows a SEM image of the morphology of the patterned surface after high-temperature annealing for 1 h at 1,000°C. Figure 4c shows the AFM image of nanopatterned Al thin films with 250-nm-wide lines separated by 550-nm space after dual-stage annealing for 24 h at 450°C and 1 h at 1,000°C. Using this technique, it can be seen that the upper surfaces of the patterns are not flat; instead, the center of the patterns is higher than the edges. Moreover, the height of the patterns following the high-temperature annealing of 1 h at 1,000°C was approximately150 nm. Our experimental results reveal that the consistency of line patterns fabricated by dual-stage annealing of patterned Al thin films for 24 h at 450°C and 1 h at 1,000°C and the orientation were the same as those of the sapphire (0001) substrates [14].

Therefore, it is believed that the above process has potential for the large-scale fabrication of NPSS for high output power GaN-based light-emitting diodes.

## Conclusions

In this study, large-scale NPSS were fabricated by dual-stage annealing of patterned Al thin films prepared by soft UV-NIL and RIE. The soft mold with 550-nm-wide lines separated by 250-nm space was composed of the toluene-diluted PDMS layer supported by the soft PDMS. The nanoimprint pressure is 3 × 10^4^ Pa, and the hold time of UV exposure is 90 s. Patterned Al thin films were subsequently subjected to dual-stage annealing. The first comprised a low-temperature oxidation anneal, where the annealing temperature was 450°C for 24 h. This was followed by a high-temperature annealing in the range of 1**,**000°C to 1,200°C for 1 h to induce growth of the underlying sapphire single crystal to consume the oxide layer. The SEM results indicate that the patterns were retained on sapphire substrates after high-temperature annealing at less than 1,200°C. Finally, large-scale nanopatterned sapphire substrates were successfully fabricated by annealing of patterned Al thin films for 24 h at 450°C and 1 h at 1,000°C by soft UV-nanoimprint lithography. It is believed that the above process has potential for the large-scale fabrication of NPSS for high output power GaN-based light-emitting diodes.

## Competing interests

The authors declare that they have no competing interests.

## Authors' contributions

LC fabricated the large-scale nanopatterned sapphire substrates by annealing of patterned Al thin films by soft UV-nanoimprint lithography, analyzed the results, and wrote and revised the manuscript. J-CH, G-GW, and HYZ participated in the revision of the manuscript. RS and L-HL participated in the preparation of Al thin films. All authors read and approved the final manuscript.

## References

[B1] Schubert EF Light-Emitting Diodes 2003 Cambridge: Cambridge University Press 19 20

[B2] Usui A Sunakawa H Sakai A Yamaguchi AA Thick GaN epitaxial growth with low dislocation density by hydride vapor phase epitaxy Jpn J Appl Phys 1997 36 L899 L902 10.1143/JJAP.36.L899

[B3] Iwaya M Takeuchi T Yamaguchi S Wetzel C Amano H Akasaki I Reduction of etch pit density in organometallic vapor phase epitaxy-grown GaN on sapphire by insertion of a low-temperature-deposited buffer layer between high-temperature-grown GaN Jpn J Appl Phys 1998 37 L316 L318 10.1143/JJAP.37.L316

[B4] Huh C Lee KS Kang EJ Park SJ Improved light-output and electrical performance of InGaN-based light-emitting diode by microroughening of the p-GaN surface J Appl Phys 2003 93 9383 9385 10.1063/1.1571962

[B5] Yamada M Mitani T Narukawa Y Shioji S Niki I Sonobe S Deguchi K Sano M Mukai T InGaN-based near-ultraviolet and blue-light-emitting diodes with high external quantum efficiency using a patterned sapphire substrate and a mesh electrode Jpn J Appl Phys 2002 41 L1431 L1433 10.1143/JJAP.41.L1431

[B6] Feng ZH Lau KM Enhanced luminescence from GaN-based blue LEDs grown on grooved sapphire substrates IEEE Photon Technol Lett 2005 17 1812 1814

[B7] Li Z Jiang Y Yu T Yang Z Tao Y Jia C Chen Z Yang Z Zhang G Analyses of surface temperatures on patterned sapphire substrate for the growth of GaN with metal organic chemical vapor deposition Appl Surf Sci 2011 257 8062 8066 10.1016/j.apsusc.2011.04.099

[B8] Gao H Yan F Zhang Y Li J Zeng Y Wang G Fabrication of nano-patterned sapphire substrates and their application to the improvement of the performance of GaN-based LEDs J Phys D Appl Phys 2008 41 115106-1 115106-5

[B9] Hersee SD Zubia D Sun X Bommena R Fairchild M Zhang S Burckel D Frauenglass A Brueck SRJ Nanoheteroepitaxy for the integration of highly mismatched semiconductor materials IEEE J Quantum Electron 2002 38 1017 1028 10.1109/JQE.2002.800987

[B10] Zang KY Wang YD Chuaa SJ Wang LS Nanoscale lateral epitaxial overgrowth of GaN on Si (111) Appl Phys Lett 2005 87 193106-1 193106-3

[B11] Nakamura S Mukai T Senoh M Candela-class high-brightness InGaN/AlGaN double-heterostructure blue-light-emitting diodes Appl Phys Lett 1994 64 1687 1689 10.1063/1.111832

[B12] Yan F Gao H Zhang Y Li J Zeng Y Wang G Yang F High-efficiency GaN-based blue LEDs grown on nano-patterned sapphire substrates for solid-state lighting Proc SPIE 2007 6841 684103-1 684103-7

[B13] Park H Chan HM Vinci RP Patterning of sapphire substrates via a solid state conversion process J Mater Res 2005 20 417 423 10.1557/JMR.2005.0050

[B14] Cui L Wang G-G Zhang H-Y Han J-C Effect of exposure parameters and annealing on the structure and morphological properties of nanopatterned sapphire substrates prepared by solid state reaction Ceram Int 2013 doi:10.1016/j.ceramint.2013.09.016

[B15] Luo G Maximov I Adolph D Graczyk M Carlberg P Ghatnekar-Nilsson S Hessman D Zhu T Liu ZF Xu HQ Montelius L Nanoimprint lithography for the fabrication of interdigitated cantilever arrays Nanotechnol 2006 17 1906 1910 10.1088/0957-4484/17/8/017

[B16] Glinsner T Plachetka U Matthias T Wimplinger M Lindner P Soft UV-based nanoimprint lithography for large-area imprinting applications Proc SPIE 2007 6517 651718-1 651718-7

[B17] Koo N Plachetka U Otto M Bolten J Heong J Lee ES Kurz H Improved mold fabrication for the definition of high quality nanopatterns by soft UV-nanoimprint lithography using diluted PDMS material Microelectron Eng 2007 84 904 908 10.1016/j.mee.2007.01.017

[B18] Ericson F Kristensen N Schweitz J A transmission electron microscopy study of hillocks in thin aluminum films J Vac Sci Technol B 1991 9 58 63 10.1116/1.585790

[B19] Maruyama T Komatsu W Surface diffusion of single-crystal Al_2_O_3_ by scratch-smoothing method J Am Ceram Soc 1975 58 338 339 10.1111/j.1151-2916.1975.tb11494.x

[B20] Bennison SJ Harmer MP Diffusion in sapphire and the role of magnesia in the sintering of alumina J Am Ceram Soc 1990 73 833 837 10.1111/j.1151-2916.1990.tb05122.x

[B21] Glaeser AM Ceramic Interfaces: Properties and Applications 1998 London: Institute of Materials 241

[B22] Bonzel HP Surface morphologies: transient and equilibrium shapes Interface Sci 2001 9 21 34 10.1023/A:1011210627335

[B23] Mullins WW Flattening of a nearly plane solid surface due to capillarity J Appl Phys 1959 30 77 83 10.1063/1.1734979

[B24] Bonzel HP Mullins WW Smoothing of perturbed vicinal surfaces Surf Sci 1996 350 285 300 10.1016/0039-6028(95)01111-0

